# Assessing the Diagnostic Value of Mean Monocyte Volume and Hematological Parameters in Predicting Dengue Fever: A Cross-Sectional Analysis

**DOI:** 10.7759/cureus.75174

**Published:** 2024-12-05

**Authors:** Nor Hayati Ismail, Alaa Siddig, Nor Azah Farhah Ab Aziz, Marini Ramli, Zefarina Zulkafli, Muhammad Farid Johan, Siti Asma Hassan, Rosnah Bahar, Noor Haslina Mohd Noor, Shafini Mohamed Yusoff

**Affiliations:** 1 Department of Hematology, School of Medical Sciences, Universiti Sains Malaysia, Kota Bharu, MYS; 2 Department of Pathology, School of Medical Sciences, Universiti Sains Malaysia, Kota Bharu, MYS; 3 Department of Pathology, Hospital Tanah Merah, Kelantan, MYS; 4 Department of Medical Microbiology and Parasitology, School of Medical Sciences, Universiti Sains Malaysia, Kota Bharu, MYS

**Keywords:** dengue fever, dengue serology, hematological parameters, igm and ns1, leukopenia, mean monocyte volume, monocytosis, platelet count, thrombocytopenia, white blood cells count

## Abstract

Background and aim: Distinguishing dengue fever (DF) from other viral infections solely based on common presentations poses a challenge. Therefore, there is a pressing need for additional diagnostic parameters that are reliable, swift, and cost-effective. This study aims to provide novel insights into the diagnostic value of hematological parameters, particularly mean monocyte volume (MMV), in predicting DF in Kelantan, Malaysia.

Methodology: This cross-sectional study enrolled 162 patients with suspected DF symptoms. The diagnosis was confirmed through dengue immunoglobulin M (IgM) capture enzyme-linked immunosorbent assay (ELISA) or Dengue Early ELISA for nonstructural protein 1 (NS1) antigen detection. Hematological parameters were measured using the Coulter DxH 800 hematology analyzer (Beckman Coulter, Brea, CA), and the statistical analysis was performed using SPSS version 22 (IBM Corp., Armonk, NY).

Results: A total of 108 patients tested positive for DF, while 54 tested negative. We observed significant differences in WBC count, platelet count, and monocyte percentage between patients with DF and non-DF, while no significant correlation was noted for MMV. Subsequent statistical analysis, including receiver operating characteristic (ROC) curve analysis, revealed that monocyte percentage exhibited the largest area under the curve (0.715), indicating its potential as moderate discriminative power in diagnosing DF.

Conclusions: Our study findings indicate that monocyte percentage and MMV outcomes are insufficient for predicting DF, suggesting potential areas for further research.

## Introduction

The prevalence of dengue fever (DF) remains a significant global health issue [1]. DF presents a wide array of symptoms, making precise laboratory diagnosis crucial due to its variable clinical features and diagnostic challenges [2]. Therefore, identifying signs of progression toward severe illness is vital for timely treatment.

Hematological indicators are essential for diagnosing and treating DF as they help identify probable cases, evaluate disease severity, and track patient response to treatment [3]. For instance, monitoring white blood cell (WBC) levels can aid in detecting potential dengue cases, especially when combined with other clinical signs. Additionally, leukopenia is associated with severe cases of dengue, and a significant decrease in WBC count can indicate the progression to severe manifestations such as dengue hemorrhagic fever (DHF) [4].

In addition, monocytes are pivotal in the immune response, undergoing significant structural and functional changes during infections. Mean monocyte volume (MMV) and standard deviation of monocyte volume (MVSD) are critical hematologic parameters that reflect the average size and variability of monocytes, respectively [5]. These measurements, obtained through automated hematology analyzers, are instrumental in diagnosing and monitoring infections, inflammatory conditions, and hematologic disorders. By providing detailed insights into monocyte characteristics, MMV and MVSD contribute to a comprehensive evaluation of a patient's hematologic status.

MMV and various hematological parameters have garnered attention for their potential role in assessing DF, particularly regarding disease severity and prognosis [5]. Although MMV is not routinely utilized in clinical practice for the diagnosis of dengue, it has been investigated in research settings as a promising biomarker for distinguishing dengue from other febrile illnesses [5].

Changes in MMV, alongside other hematological parameters such as platelet counts and leukocyte differentials, can provide valuable insights into the patient's immune response and disease progression, that are routinely employed in the diagnosis and management of dengue [6]. In addition, elevated MMV may indicate increased monocyte activity, which is associated with the inflammatory responses characteristic of DF [6].

In the context of dengue virus (DENV) infection, monocytes are primary targets in peripheral blood mononuclear cells, and their activation has been linked to DENV pathogenesis [7]. Infected monocytes may exhibit changes in size and granularity, which can be detected through variations in MMV observed in a complete blood count (CBC) [8]. This is supported by findings indicating that patients with DF in the early stages, especially those with a high viral load, have elevated monocyte levels. These alterations in the peripheral circulation are associated with modifications in the monocyte population [9]. Overall, variations in monocyte size or function can influence the MMV in a blood sample, serving as a predictive measure for dengue infection [8]. This fact underscores the importance of MMV and MVSD as valuable hematologic parameters in the assessment and management of infectious diseases.

Given the rapid progression of DF, particularly in severe cases, early detection of immune system alterations is critical for timely intervention. Therefore, MMV may serve as an early biomarker for abnormal immune responses, thereby enhancing the prognostic accuracy of clinical assessments and enabling more effective management of the disease. In this study, we aim to evaluate the potential of MMV as a diagnostic marker for our patients diagnosed with DF and compare its performance with other hematological parameters. The implications of this study can be used to improve dengue diagnosis, enhance patient management strategies, and contribute to the existing knowledge base on dengue pathophysiology.

## Materials and methods

**Figure 1:**
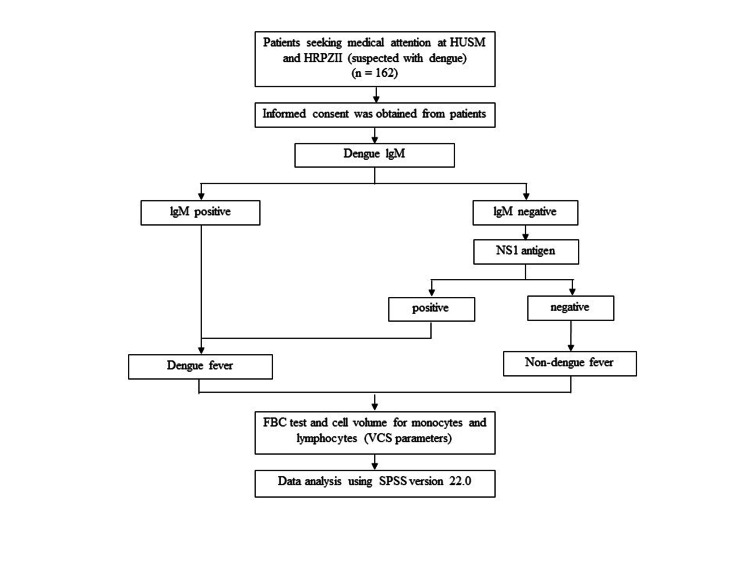
The overview of the flowchart outlining the sequential steps and procedures in this study. HUSM, Hospital Universiti Sains Malaysia; HRPZII, Hospital Raja Perempuan Zainab II; FBC, full blood count; VCS, volume, conductivity, and scatter; NS1, nonstructural protein 1

Figure 1 summarizes the methodology employed in this study visually. It presents a flowchart outlining the sequential steps and procedures followed to achieve the study objectives.

Study design

The cross-sectional study included 162 patients clinically suspected of DF and was conducted at Hospital Universiti Sains Malaysia (HUSM) and Hospital Raja Perempuan Zainab II (HRPZII), Kelantan, Malaysia. The study was conducted from August 1, 2013, to November 27, 2014, and was approved by the School of Medical Science Research Ethical Committee, USM (protocol number: USM/JEPeM/140379). Written consent was obtained from all study participants.

Inclusion criteria comprised patients clinically suspected of DF aged 17 years and above, encompassing all races and genders. Conversely, patients below 17 years old or those aged 17 and above with underlying hematological disorders, autoimmune diseases, or confirmed alternative sources of infection were excluded. The control group was included in this study and consisted of healthy blood donors (*n* = 30) from the blood bank unit at HUSM.

A systematic random sampling method was employed by selecting every third patient from the patient list until the required sample size was attained. In addition, the demographic data, including age, gender, and clinical history, were obtained by reviewing the medical records of all study participants.

Sample collection and processing

Three milliliters of whole blood were collected into tubes containing ethylenediaminetetraacetic acid (EDTA) for hematological parameters and plain tubes for dengue serology tests. The following hematological parameters were analyzed using the DxH 800 hematology analyzer (Beckman Coulter, Brea, CA): WBC count, platelet (PLT) count, hematocrit (HCT), differential count (DC), and volume, conductivity, and scatter (VCS) parameters of monocyte, including MMV and MVSD.

For serology testing, serum samples were separated and processed using the Panbio™ Dengue IgM Capture enzyme-linked immunosorbent assay (ELISA) and Panbio Dengue Early ELISA kits to detect the presence of dengue IgM and nonstructural protein 1 (NS1) antigen, respectively. The testing procedures were carried out according to the manufacturer's protocol. Results from both tests were interpreted as negative, equivocal, or positive based on the outcomes index obtained from the ELISA testing.

Statistical analysis

The results were analyzed using the statistical package program SPSS version 22.0 (IBM Corp., Armonk, NY). Standard hematological parameters and VCS parameters were subjected to univariable analyses, utilizing independent t-tests to compare two means. A *P*-value < 0.05 was considered statistically significant. Descriptive statistics were employed for numerical variables, expressed as mean and standard deviation (SD) for data following a normal distribution, and for categorical variables, expressed as frequency and percentage. Receiver operating characteristic (ROC) analysis was utilized to assess the discriminant ability of VCS parameters in detecting DF. The ROC curve is created by plotting the true positive rate (sensitivity) against the false positive rate (1 - specificity) at various threshold settings.

## Results

Demographic characteristics of study subjects

Out of the 162 patients recruited, 108 (66.7%) cases were confirmed as DF, while 54 (33.3%) were non-DF. The mean ages of patients with DF and non-DF cases were reported as 35.31 years and 39.02 years, respectively. In the DF group, a higher infection rate was demonstrated in male patients (59, 54.6%) than female patients (59, 45.4%). However, the study found an almost similar gender distribution in non-DF patients. On the other hand, this study found no statistically significant difference (*P* < 0.05) in age, gender, or ethnic group between DF and non-DF. The details of the demographic characteristics of study subjects are illustrated in Table 1.

**Table 1:** Comparing patients’ demographic data between dengue and non-dengue cases. Age was presented in the form of mean ± SD, whereas gender and race were presented in the form of frequency and percentage. To assess the statistical difference in the distribution of cases between the two groups, an independent t-test and chi-square test were used. A *P*-value of <0.05 was considered significant. ^a^*P*-value for the independent t-test. ^b^*P*-value for the chi-square test.

Demographic characteristic	Dengue cases (n = 108)	Non-dengue cases (n = 54)	*P*-value
Age (years), Mean ± SD	35.31 ± 14.1	39.02 ± 16.4	0.139^a^
Gender, n (%)			
Male	59 (54.6)	26 (48.1)	0.245^b^
Female	49 (45.4)	28 (51.9)	
Race, n (%)			
Malay	102 (94.4)	50 (92.6)	0.509^b^
Non-Malay	6 (5.6)	4 (7.4)	

Dengue serology in DF patients

A greater proportion of patients with DF tested positive for the NS1 antigen. Specifically, 68 (63%) patients showed the presence of this antigen. On the other hand, only 40 (37%) DF patients tested positive for the IgM antibody.

Hematological parameters

This study highlights several abnormal hematological parameters in DF patients. Thrombocytopenia was the most common presentation in DF patients, which was observed in 72 (66.7%) patients, followed by monocytosis (59, 54.6%), leukopenia (50, 46.3%), and lymphocytosis (28, 25.9%). However, this study found that only one patient (0.9%) exhibited hemoconcentration. Table 2 provides an overview of these abnormal hematological conditions resulting from changes in hematological parameters observed in the study.

**Table 2:** Abnormal hematological condition and parameters in DF patients (n = 108). The abnormal hematological parameters were presented in frequency and percentage. PLT, platelet; WBC, white blood cells

Hematological parameters	*n* (%)	Normal range
Leukopenia	50 (46.3)	WBC < 4.0 x 10^9^/L
Thrombocytopenia	72 (66.7)	Platelet < 150 x 10^9^/L
Hemoconcentration	1 (0.9%)	Men: <0.45 +/- 0.05; women: <0.41 +/- 0.05
Monocytosis	59 (54.6)	2%-10%
Lymphocytosis	28 (25.9)	>40%

Comparison of standard hematological parameters between DF patients and non-DF patients

Differences in mean standard hematological parameters between DF and non-DF patients are detailed in Table 3. Initially, WBC, HCT, and PLT counts showed a decreasing trend, while monocyte (%) and lymphocyte (%) exhibited an increasing trend in DF patients. However, our analysis revealed that only WBC and PLT counts were significantly lower (*P* < 0.05) in DF patients compared to non-DF patients. Conversely, monocyte (%) showed a significant increase in DF patients. Additionally, we found that mean HCT levels were similar in both groups, with no significant association observed in this parameter in DF patients.

**Table 3:** Comparing the mean standard hematological parameters in DF and non-DF dengue patients (n = 162). Data were presented as mean ± SD; a t-test was used to compare the mean value between the two comparison groups. A *P*-value less than 0.05 was considered significant. PLT, platelet; WBC, white blood cells; HCT, hematocrit; SD, standard deviation

Hematological parameters	DF (*n *= 108), mean ± SD	Non-DF (*n* = 54), mean ± SD	Mean difference (95% CI)	t-statistic (df)	*P*-value
WBC (x10^9^/L)	4.51 ± 2.26	6.86 ± 3.40	-2.34 (-3.36, -1.32)	-4.58 (77)	0.000
HCT (L/L)	40.56 ± 5.03	40.70 ± 5.52	-0.14 (-1.85, 1.57)	-0.16 (160)	0.874
PLT (x10^9^/L)	123.04 ± 78.78	177.61 ± 72.17	-54.57 (-79.81, -29.34)	-4.27 (160)	0.000
Monocyte (%)	11.82 ± 9.40	7.56 ± 6.65	4.29 (1.46, 7.11)	3.00 (160)	0.003
Lymphocyte (%)	30.13 ± 13.49	27.29 ± 15.14	2.84 (-1.79, 7.47)	1.21 (160)	0.228

Comparison of monocyte and lymphocyte volume between DF patients and and the normal population 

In this study, patients with DF exhibited higher mean monocyte and lymphocyte volumes compared to the normal population, and these differences were statistically significant (*P* < 0.05), as illustrated in Table 4.

**Table 4:** Comparing the mean monocyte and lymphocyte volume in DF patients and normal population (n = 128). Data were presented as mean ± SD; a t-test was used to compare the mean value between the two comparison groups. *P*-value < 0.05 was considered significant. MMV, mean monocyte volume; MVSD, monocyte volume SD; MLV, mean lymphocyte volume

Parameters	DF (*n* = 108), mean ± SD	Normal population (*n* = 20), mean ± SD	Mean diff. (95% CI)	t-statistic (df)	*P*- value
MMV	205.4 ± 15.85	179.2 ± 6.9	26.1 (19.0-33.3)	7.2 (126)	0.000
MVSD	29.9 ± 4.81	23.7 ± 3.2	6.14 (4.4-7.9)	7.3 (38)	0.000
MLV	92.9 ± 11.15	87.1 ± 5.9	5.84 (3.9-8.4)	3.5(49)	0.001

Table 5 indicates that patients with DF had slightly higher MMV, MLV, and LVSD, but lower MVSD compared to non-DF patients. Therefore, we did not find significant differences in monocyte and lymphocyte volumes between cases of DF and non-DF patients.

**Table 5:** Comparing mean monocyte and lymphocyte volume in DF and non-DF patients (n=162) Data were presented as mean ± SD; a t-test was used to compare the mean value between the two comparison groups. *P*-value < 0.05 was considered significant. MMV, mean monocyte volume; SD, standard deviation; MVSD, monocyte volume SD; MLV, mean lymphocyte volume; LVSD, lymphocyte volume SD

Parameters	DF (*n* = 108), mean ± SD	Non-DF (*n* = 54), mean ± SD	Mean diff. (95% CI)	t-statistic (df)	*P*-value
MMV	205.34 ±16.09	203.17 ± 14.35	2.19 (-2.70, 7.07)	0.89 (119)	0.378
MVSD	29.94 ± 4.90	30.01 ± 6.42	-0.08 (-1.86, 1.72)	-0.13 (160)	0.939
MLV	93.39 ±11.25	90.46 ± 16.56	2.93 (-1.43, -7.29)	1.33 (160)	0.187
LVSD	27.54 ± 7.52	26.36 ± 7.84	1.18 (-1.33, 3.69)	0.93 (160)	0.354

ROC analysis

**Figure 2:**
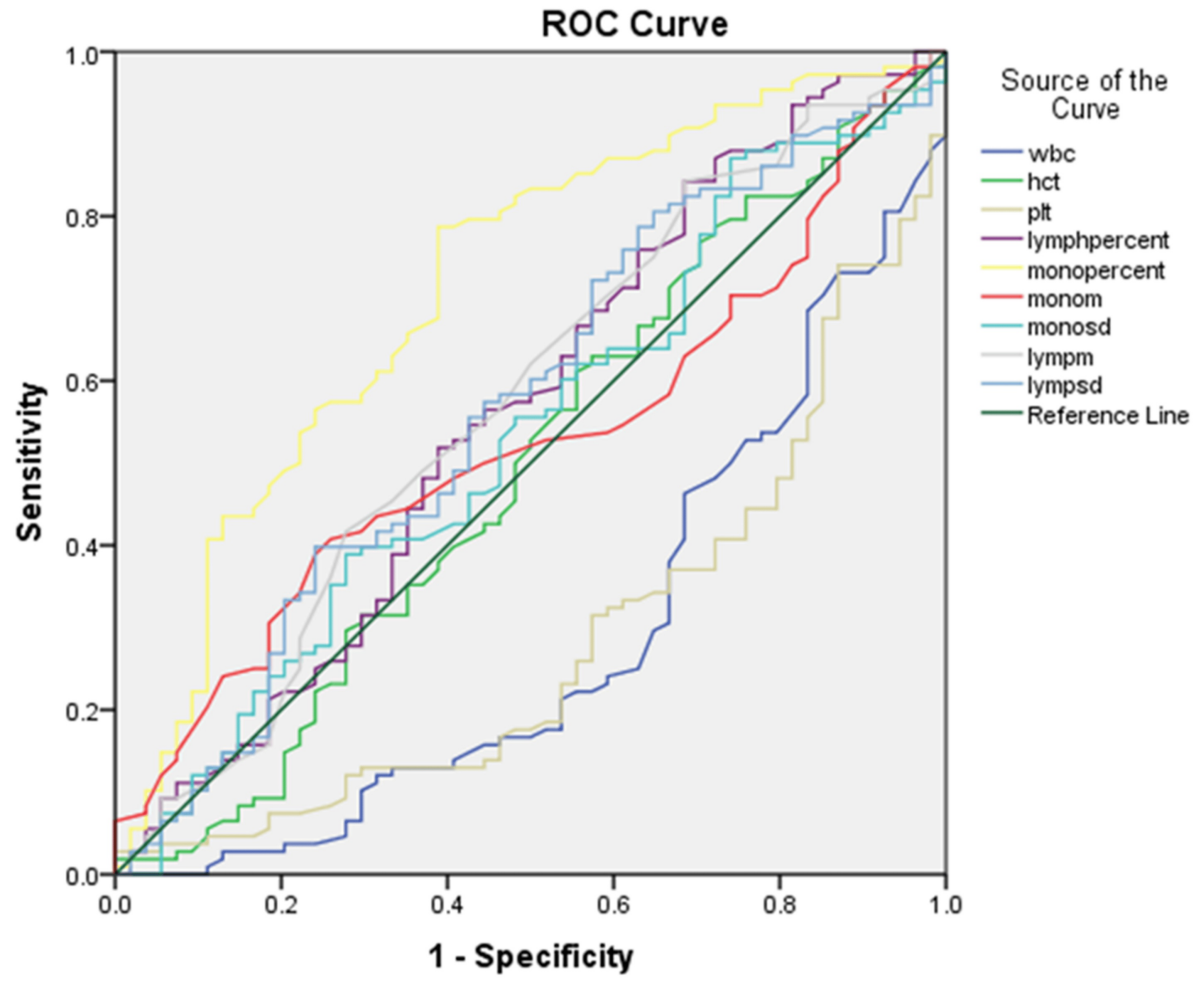
Generated ROC curves for optimal cutoff values of hematological parameters in predicting DF. WBC, white cell count; Hct, hematocrit; PLT, platelet; lymp, lymphocyte; mono, monocyte; monom, mean monocyte volume; monosd, monocyte volume standard deviation; lymphm, mean lymphocyte volume; lympsd, lymphocyte volume standard deviation

ROC curves were generated to determine the optimal cutoff values of hematological parameters for predicting DF. Parameters including monocyte volume, lymphocyte volume, WBC count, hematocrit, monocyte and lymphocyte percentages, and PLT counts were analyzed to determine their area under the curve (AUC). Figure 2 illustrates the ROC comparisons of these hematological parameters for predicting DF.

The results show that monocyte percentage had the largest AUC (0.715), with a sensitivity of 98.1% and specificity of 92.6% at the lowest cutoff value of 1%, outperforming other standard hematological parameters such as MLV, LVSD, MMV, and MVSD. High sensitivity corresponding to that monocyte percentage correctly identifies a high proportion of true DF cases among non-DF. Additionally, the high specificity reflects that monocyte percentage correctly identifies a high proportion of true non-DF cases among those non-DF. This result indicates that monocyte percentage is a moderately good predictor for distinguishing between patients with DF and non-DF, which is better than a random guess (AUC = 0.5). The details of AUC, cutoff points, sensitivity (%), and specificity (%) for each hematological parameter are provided in Table 6.

**Table 6:** Predicting DF utilizing different hematological parameters. Data were presented as sensitivity (%) and specificity (%) of AUC values between hematological parameters. PLT, platelet; WBC, white blood cells; HCT, hematocrit; MMV, mean monocyte volume; SD, standard deviation; MVSD, monocyte volume SD; MLV, mean lymphocyte volume; LVSD, lymphocyte volume SD

Parameters	AUC	Cutoff points	Sensitivity (%)	Specificity (%)
PLT	0.287	33.5	89.8	98.1
WBC	0.287	2.05	88	98
HCT	0.497	29.25	98	98
Monocyte (%)	0.715	1.0	98.1	92.6
Lymphocyte (%)	0.562	5.3	99.1	96.3
MMV	0.527	172.50	98.1	98.1
MVSD	0.534	21.17	96.3	98.1
MLV	0.574	63.50	99.1	98.1
LVSD	0.568	15.28	98.1	98.1

## Discussion

In this study, we aimed to assess the distribution of hematological parameters between patients infected with DENV and those who were not. Among the 162 patients suspected of DENV infection, 108 (66.7%) were confirmed to have DF. The mean age of patients with DF was 35.3 years, closely aligning with previous research conducted among Indian patients, which reported a mean age of 35.9 years [10]. Notably, studies consistently demonstrate that dengue is more prevalent among younger populations compared to older demographics [11].

In the present study, a significant proportion (*P* < 0.05) of DF patients (68, 63%) tested positive for NS1, strongly suggesting that they were in the early stages of infection [12]. Only one-third of DF patients tested positive for IgM, which still indicates a recent infection compared to those who tested positive for IgG. An earlier study involving more than 400 patients reported that the presence of dengue NS1 antigen could be identified as late as day 12 from the onset of fever, whereas reverse transcription polymerase chain reaction (RT-PCR) detects viral RNA only up to day 8 post-infection. This suggests that NS1 antigen testing may be more effective for diagnosing DF by providing a longer window for accurate diagnosis compared to RT-PCR [13].

Detecting IgM and IgG antibodies in both acute and recovering patients involves conducting ELISA tests. The IgM Antibody Capture ELISA specifically detects IgM antibodies, while the indirect IgG ELISA is used for IgG detection [14]. The presence of IgM antibodies indicates a recent or ongoing infection with DENV. IgM antibodies typically appear within the first week of symptom onset and peak during the acute phase of the illness, making them valuable for identifying acute dengue infection [15].

Moreover, the identification of IgG antibodies signifies the probability of previous contact with the DENV among our study subjects. IgG antibodies are produced at a later stage of infection and remain in the body for extended periods, offering long-lasting protection against different strains of the DENV. A positive IgG test indicates prior infection with the DENV or vaccination against it [15].

Notably, the condition of coexistence of IgM and IgG antibodies may be detected in DF patients, which may indicate a subsequent dengue infection. During secondary infections, the IgG antibodies produced from a prior infection interact with the DENV serotype responsible for the present illness. This interaction triggers a quick and strong response from the IgM antibodies [16]. Subsequently, the antibody-dependent enhancement can lead to the emergence of severe symptoms of dengue, such as DHF or dengue shock syndrome (DSS) [16].

An in-depth investigation of the hematological laboratory tests of DF patients showed that 72 (66.7%) DF patients had thrombocytopenia, which is a common finding in DENV-infected patients. However, the exact mechanism involved in the thrombocytopenia is not fully clear. As an extension to our findings, Sami et al. revealed that the combination of thrombocytopenia, hypotension, and hemoconcentration during the febrile phase may indicate an increased risk of progression toward severe disease. These presentations have been observed in the latest trends of the deadliest DF in Bangladesh in 2022 [17].

DF is proposed to affect the function of bone marrow progenitor cells, resulting in lower proliferation capability [18]. Earlier studies suggest that thrombocytopenia is either the result of decreased production of PLT or an increase in the destruction and clearance of those cells from the peripheral blood [19]. If the MPV is elevated, this may mirror an increase in the destruction of the PLT [18].

The CBC shows that more than half of DF patients (59, 54.6%) exhibited monocytosis. This is in line with an earlier study conducted among Taiwanese patients (*n* = 1,015), where researchers observed that severe patients had higher absolute monocyte counts on day 5 following the onset of fever compared to individuals with mild fever, particularly in those infected with DENV2 and DENV3. They hypothesized that rapid thrombocytopenia occurs alongside a temporary increase in monocytes beginning on the fourth day, suggesting that the phagocytic function of innate immune cells plays a role in causing a thrombocytopenia state in adults with DF [20].

Additionally, the leukopenia observed in 50 (46.3%) DF patients aligns with the findings of Chaloemwong et al., who documented a decrease in WBC count starting on day 2, reaching its lowest point on day 5 of the fever, and then gradually improving [21]. This decline is believed to result from the destruction of myeloid progenitor cells, as the bone marrow witnesses suppression in the first seven days [4].

Lymphocytosis was observed in 28 (25.9%) DF patients; this percentage was lower than the percentage observed in previous studies (64% and 50%) [22]. It's well established that lymphocytes and atypical lymphocytes are elevated in DENV infection, reflecting the active dynamics of the immune system [22]. Previous studies have linked lymphocytosis in dengue infection to secondary infections also. They suggest that when non-neutralizing antibodies from a past infection quickly interact with the virus during a second infection, these antibodies help the virus enter cells through Fc-receptors. This process activates T-lymphocytes, leading to lymphocytosis [23].

In this study, we investigated the potential of MMV as a diagnostic biomarker to distinguish between DF and non-DF patients. However, our findings demonstrate that MMV exhibited insufficient discriminative power in differentiating DF patients from those with other febrile illnesses or non-DF conditions. This result suggests that MMV alone may not be a reliable diagnostic tool for DF, contrary to previous studies that have suggested MMV could serve as a marker of immune activation during viral infections, including dengue [6]. For example, Poottasane et al. reported that monocyte distribution width which measures the variation in the volume of monocytes significantly increases in dengue infections and can be easily integrated into a predictive scoring system for dengue, offering a promising tool for early diagnosis and management [6].

Several factors may account for the limited diagnostic utility of MMV observed in our cohort. First, the heterogeneity of the immune response in dengue infections could contribute to the lack of distinct monocyte volume changes [24]. While monocyte activation and morphological alterations are well-documented features of viral infections, the degree of monocyte enlargement is likely to be influenced by a variety of factors, including the severity of the disease, the immune status of the individual, and the presence of co-infections [25]. For example, other viral or bacterial infections with overlapping clinical features, such as chikungunya, zika, or typhoid fever, could induce similar monocyte changes, leading to false positives and reducing the specificity of MMV for dengue [26].

In addition, dengue is a clinically diverse disease, ranging from asymptomatic to severe forms, such as DHF and DSS. MMV may be more reflective of the severity of the disease, particularly in severe cases, where profound immune activation and inflammation are more pronounced. In contrast, in mild cases of DF, the changes in monocyte size may be less distinct, leading to poor differentiation between DF and non-DF patients [27]. This could explain the low sensitivity and specificity observed in our analysis, especially for patients with less severe or non-specific symptoms.

Furthermore, the technical variability in MMV measurement should be considered. Factors such as the timing of blood sample collection, variations in laboratory protocols, and the use of different diagnostic platforms could all contribute to inconsistencies in MMV values [28]. The dynamic nature of the immune response in dengue, where monocyte activation and volume may fluctuate throughout infection, could further complicate the interpretation of MMV as a diagnostic marker [29]. Additionally, the cutoff thresholds for MMV that would distinguish DF from non-DF patients may vary across different populations and settings [8], underscoring the need for standardization in MMV measurement.

## Conclusions

In conclusion, while MMV did not demonstrate sufficient discriminative power to differentiate DF from non-DF patients in this study, it should not be represented as a potential biomarker. As a result, our study suggests that relying solely on monocyte percentage for diagnosis is inadequate. Future research should focus on combining monocyte percentage with other biomarkers or clinical features to improve diagnostic accuracy. A broader prospective study is warranted to explore these combinations further and validate their effectiveness in differentiating DF from other febrile illnesses. Such research could lead to significant improvements in laboratory workflows and, consequently, enhance patient care quality in clinical settings.
